# Professional dental care and survival rates in long‐term care recipients: A cohort study of 1 459 163 individuals in South Korea

**DOI:** 10.1111/ger.12781

**Published:** 2024-07-30

**Authors:** Kyung‐A Ko, Bo‐A Lee, Young‐Taek Kim, Jung‐Seok Lee

**Affiliations:** ^1^ Department of Periodontology Research Institute of Periodontal Regeneration, Yonsei University College of Dentistry Seoul Korea; ^2^ Innovation Research and Support Center for Dental Science Yonsei University Dental Hospital Seoul Korea; ^3^ Department of Periodontology National Health Insurance Service Ilsan Hospital Goyang Korea

**Keywords:** institutionalised, long‐term care, older adults, oral health

## Abstract

**Objectives:**

To determine the dental care pattern and survival rates of participants who received long‐term care (LTC) and a matched control cohort in South Korea.

**Background:**

Global ageing trends and the development of superaged societies pose healthcare challenges. South Korea's LTC system aids those with chronic illnesses and disabilities. Despite the link between oral health and systemic diseases, providing dental care in LTC facilities often reflects social neglect.

**Methods:**

We identified 1 459 163 individuals eligible for LTC insurance in the Korean National Health Insurance Service database from July 2008 to 2015 (LTC cohort) and 1 459 544 individuals matched through propensity‐score matching (matched cohort). The LTC recipients were further categorised into subgroups based on their care type (institutional, home or mixed care). Population of utilising dental services and the average number of dental visits were counted in each cohort, and the survival rate of the LTC cohort was determined according to dental utilisation.

**Results:**

Population of utilising dental services increased steadily in all cohorts except for institutional care, with the highest utilisation (around 30%) observed in the matched cohort. Lower independence in LTC cohorts was associated with lower dental utilisation: 18‐27% for home care, 12‐18% for mixed care, and 10% for institutional care. The survival rates in the LTC cohort were significantly lower than in the matched cohort (*P* < .0001), with 28.1% survival in LTC vs 59.3% in the matched cohort.

**Conclusion:**

Long‐term care recipients experience social neglect for oral care, while higher survival rates were observed in those utilised dental services.

## 1 INTRODUCTION

**FIGURE 1:**
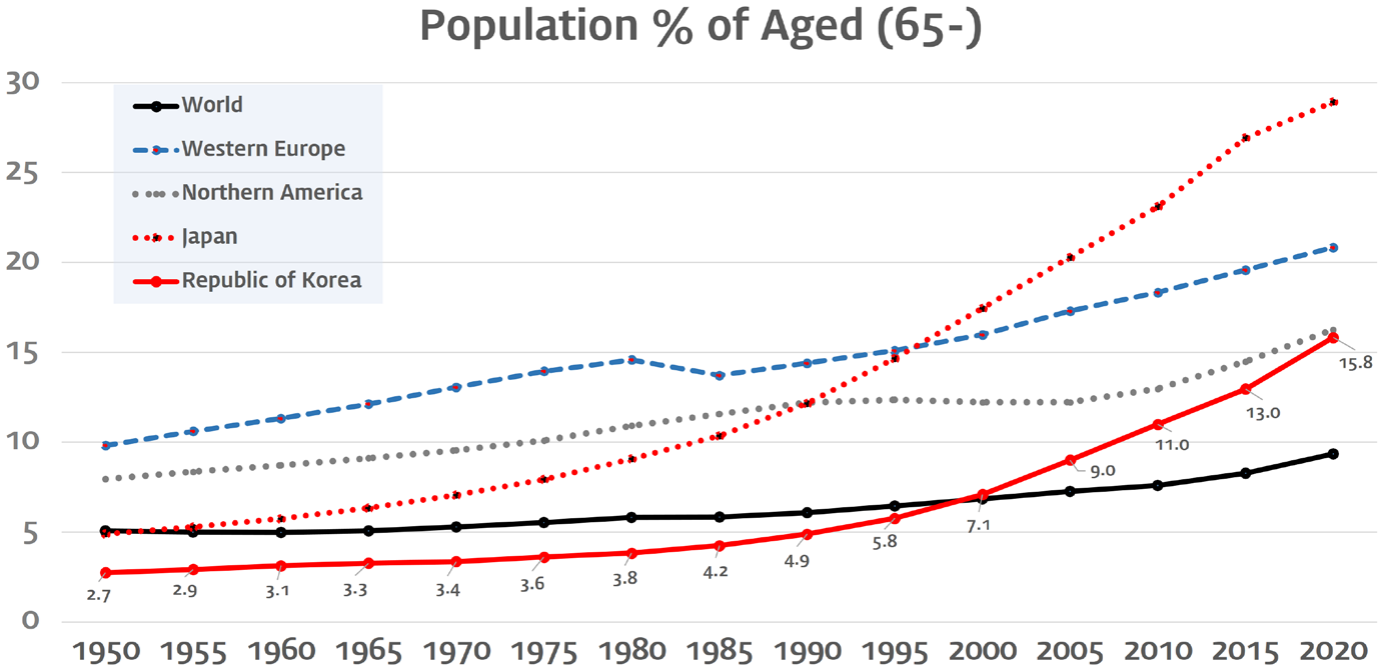
Ageing population chart from 1950 to 2021, based on data from the UN (https://population.un.org/wpp/), displays a solid red line representing a significant upward trend in the proportion of the population aged over 65 in South Korea. This line illustrates the rapid progression towards a super‐aged society. [Colour figure can be viewed at wileyonlinelibrary.com]

The rising proportion of the older population within a society correlates with a growing prevalence of medical/dental healthcare challenges. Worldwide life expectancy has been dramatically increasing during the last 100 years, and it has led to a progressive ageing of the population. In particular, many developed countries became or are becoming superaged societies, defined as >20% of the population being aged ≥65 years. As the oldest superaged country, the older population of Japan accounts for almost one‐third of its entire population (28.40%), and the countries of western Europe can also considered to be in the same category, with older people accounting for 20.84% of their population in 2020.^1^ South Korea has one of the fastest‐increasing proportions of older people along with Japan, and is expected to become a superaged society within 5 years (Figure 1).^2^ While the modern superaged society is witnessing a rise in a healthy and active ‘new older people’ population, a significant portion of the older demographic exhibits a higher prevalence of disease and dependency. Most developed/developing countries are therefore increasing support for this population through the implementation of a national service for long‐term care (LTC).^3^, ^4^

Long‐term care is a social service designed to support individuals with chronic illnesses and disabilities in their daily lives, offering both medical and non‐medical care. While its primary focus is on older individuals with diverse disabilities, its reach extends to a broad spectrum of recipients, ranging from those in their twenties to individuals aged over 80. LTC services vary between countries according to factors such as cultural differences, funding issues and needs of the people. South Korea has provided all LTC services through the national insurance system since 2008.^5^ The South Korean LTC service includes an eligibility selection process. When a person applies for LTC benefits, the LTC level is assigned on a scale from 1 to 5 by assessing physical function, cognitive recognition function, behavioural change, medical treatment and disability. The beneficiary is provided prescribed services according to their LTC level and care type: home‐ or facility‐based.^6^ Most medical treatments for disease control in South Korea are covered by the national health insurance, and the medical histories of all individuals are added to the digital data of the insurance service. The data from a cohort of LTC recipients is expected to provide reliable evidence for describing the present status of LTC in South Korea and for evaluating the associations among various medical conditions in ageing individuals with disabilities.

Recent scientific evidence has suggested potential associations between oral health and systemic disease occurrence.^7^, ^8^ While definitive causality has not been established, some studies have indicated that non‐communicable diseases such as cardiovascular diseases,^9^, ^10^ stroke,^11^ respiratory diseases^12^ and diabetes mellitus may be associated with periodontal inflammation.^13^, ^14^ Among them, obstructive lung disease is the most common for patients in LTC, especially those with long‐term hospitalisation.^15^ Poor oral hygiene produces a large reservoir for various microbiome types, which can directly induce a microbial bolus that hospitalised patients can inhale.^16^, ^17^ Maintaining oral health is an important factor in preventing multiple systemic diseases, including lung disease.^18^ However, the availability of dental care programs is poor for patients in LTC facilities, especially for older people.^19^, ^20^ Recent studies have suggested that the oral condition of hospitalised patients declines after admission,^21^ which increases the difficulty of achieving an adequate nutrition intake, leading to a vicious cycle of overall health‐related ailments.

The present study aimed to investigate the patterns of medical/dental care use and the survival rates of LTC recipients in South Korea.

## 2 MATERIALS AND METHODS

### 2.1 Data source

The data source was a specific database for LTC insurance provided by the National Health Insurance Service (NHIS) of South Korea. The NHIS is a public database encompassing health care utilisation, health screening, socio‐demographic variables, and mortality data for the entire population of South Korea. The LTC insurance database includes information about activities of daily living and service grades. In the NHIS, de‐identified join keys replace personal identifiers to interlink these databases.^22^

The target population of LTC insurance is older people over 65 years and those younger than 65 years with a geriatric disease such as dementia, stroke or Parkinson's disease. Among these, a person is considered to be an LTC beneficiary if a specialised medical examiner and the LTC committee have determined that to have experienced difficulty in performing the activities of daily living for longer than 6 months. The LTC committee reviews the needs‐assessment questionnaire and the results of an examination performed by a physician, and finally assigns a grade to the LTC beneficiary (grades 1‐6, A, B or C, or a re‐evaluation) (Table S1). Recipients in grades 1 and 2 receive institutional care services and those in grades 3‐5 receive home care services such as home nursing, welfare devices and home service. Those with grades A, B and C receive local healthcare services, and a patient from grade A who is diagnosed with dementia is regraded to level 5 or 6.

### 2.2 Study design and population

**FIGURE 2:**
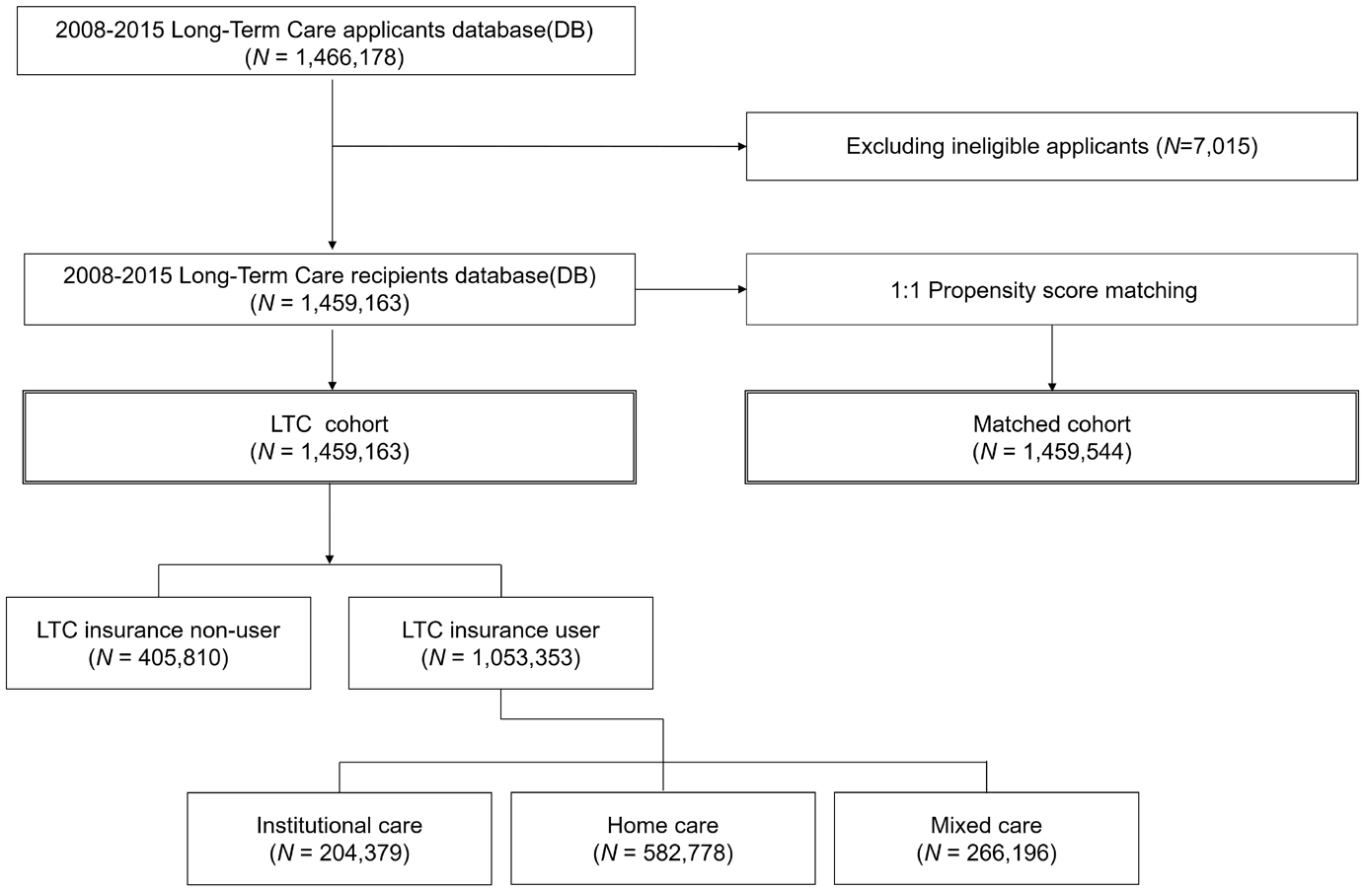
Flowchart of the cohort study design. LTC, Long‐term care.

This study included two cohorts: the beneficiary cohort that received LTC insurance from July 2008 to 2015, and the matched cohort. Among all of the applicants (*n* = 1 466 178), 1 459 163 participants were finally defined as the LTC cohort, with 7015 excluded due to them not meeting the LTC eligibility criteria. A matched cohort with the same propensity scores for sex and age as the LTC cohort was extracted from the South Korean NHIS database. The LTC cohort was divided into two groups according to the use of LTC benefits: LTC insurance users (*n* = 1 053 353; 72.2%) and LTC insurance non‐users (*n* = 405 810; 27.8%). The LTC insurance non‐users did not receive national services by their own personal choices, despite the LTC committee's determination. However, a previous report revealed that there were no significant differences in demographic characteristics between LTC users and non‐users.^23^ The LTC insurance users were further categorised into subgroups based on their care type: institutional (*n* = 204 379; 19.4%), home (*n* = 582 778; 55.3%) and mixed (*n* = 266 196; 25.3) care. The overall flowchart of this study is presented in Figure 2.

The study protocol was reviewed and approved by the Institutional Review Board (IRB) of the National Health Insurance Service Ilsan Hospital, South Korea (IRB no. NHIMC2022‐08‐007). The IRB confirmed that this study did not need to obtain informed consent because it had a retrospective design and evaluated the data collected and recorded in a way that anonymised the participants. This study used the NHIS‐NSC data (NHIS‐2020‐1‐133) and was conducted in accordance with the STROBE (Strengthening the Reporting of Observational Studies in Epidemiology) statement guidelines.

### 2.3 Data collection

All socioeconomic and medical/dental data were collected from an NHIS dataset in South Korea. A statistics specialist from the NHIS merged it with the LTC insurance data using de‐identified join keys, which replaced personal identifiers, ensuring privacy and security. The complete merged dataset was then used in an encrypted form for privacy reasons, and included the information on age, sex, income quintiles, presence of disability, type of disability, comorbid disease and dental service utilisation of each individual in both cohorts. Income quintiles were defined according to quarterly income, with the first quintile corresponding to the lowest income level. Disabilities were categorised as non‐disabled, physically disabled, disability from brain lesion and hearing/visual loss. Comorbid disease (both medical and dental conditions) prevalence was obtained from the data before LTC service inclusion, and their diagnoses were coded according to the Korean Standard Classification of Diseases, the Korean version of the International Classification of Diseases, 10th revision (ICD‐10) (Table S2). The included systemic diseases were selected based on a previous study that identified lifestyle‐related diseases related to oral health among the South Korean NHIS data^24^: cerebral infarction (ICD‐10 codes I63‐I66), angina pectoris (I20), myocardial infarction (I21, I22), hypertension (I10, I15), diabetes mellitus (E10‐E15), rheumatoid arthritis (M05, M06), erectile dysfunction (N48, N49), osteoporosis (M80‐M82) and dementia (F00).

Population of utilising dental services and the average number of dental visits were determined from the dataset by searching for diagnostic codes and dental treatment codes related to routine dental procedures. The diagnostic codes were impacted teeth (K01), dental caries (K02), another disease of hard tissue in the teeth (K03; attrition, erosion, abfraction, ankylosis, root resorption and hypercementosis), pulpitis (K04) and periodontitis (K05); the types of dental treatment were tooth extraction, endodontic treatment, periodontal treatment, implant surgery and dentures (Table S3). Survival information obtained from the NHIS database was used, and the survival of each individual was defined as the period from inclusion in the LTC service to the date of death.

The LTC and matched cohorts were compared during 2008‐2015 as follows: (1) the annual change in the population of utilising dental services, (2) the average number of visits for dental treatment, and (3) the survival rate for the LTC cohort according to whether or not dental services were utilised.

### 2.4 Statistical analysis

Statistical analysis was performed using SAS software (version 9.2, SAS Institute, Cary, North Carolina, USA). The chi‐square test was used to compare demographic characteristics, comorbid diseases, survival, dental disease and dental utilisation, and a one‐way analysis of variance was used to compare the ages of the two cohorts. The survival rates according to dental treatment in the LTC cohort were compared using a Chi‐square test, and significance was considered to be present when the two‐tailed *P*‐value was <.001.

## 3 RESULTS

### 3.1 Cohort description

Table 1 presents the baseline demographic information. Both the LTC and matched cohorts had the same age and sex distributions, and there were twice as many female recipients than male recipients. The age of recipients ranged from 20 to >80 years, with no difference in the age distribution of the two cohorts. However, most of the recipients were older than 60 years, with the highest proportion being in the 70‐79 age group. However, there were significant differences in economic status and the presence and type of disability between the LTC and matched cohorts (*P* < .0001). There was a higher proportion of participants in the lowest household income quintile in the LTC cohort (31.7%, vs. 22.7% in the matched cohort). Among all of the participants, 19.3% were disabled with more participants with any disability included in the LTC cohort. The proportions of disabilities in the LTC cohort were almost twice those in the matched cohort.

**TABLE 1:** Demographic characteristics between the long‐term care and matched cohorts.

	Long‐term care cohort (*n* = 1 459 163)		Matched cohort (*n* = 1 459 544)		Total (*n* = 2 918 707)		*P*‐value*
	*n*	*%*	*n*	*%*	*n*	*%*	
*Sex*							
Men	473 280	32.4	473 446	32.4	946 726	32.4	
Women	985 883	67.6	986 098	67.6	1 971 981	67.6	
*Age group*							
≤29	602	<.1	602	<.1	1204	.0	
30‐39	2093	.1	2093	.1	4186	.1	
40‐49	12 141	.8	12 145	.8	24 286	.8	
50‐59	47 425	3.3	47 433	3.2	94 858	3.3	
60‐69	270 556	18.5	270 633	18.5	541 189	18.5	
70‐79	645 414	44.2	748 227	51.3	1 393 641	47.7	
80≤	480 932	33.0	378 411	25.9	859 343	29.4	
*Household income* * <.0001							
First quintile	461 968	31.7	331 923	22.7	793 891	27.2	
Second quintile	134 087	9.2	147 015	10.1	281 102	9.6	
Third quintile	174 301	11.9	193 565	13.3	367 866	12.6	
Fourth quintile	250 078	17.1	283 100	19.4	533 178	18.3	
Fifth quintile	438 729	30.1	503 941	34.5	942 670	32.3	
*Type of disability* * <.0001							
Non‐disabled	1 060 556	72.7	1 294 651	88.7	2 355 207	80.7	
Physical disability	169 650	11.6	87 370	6.0	257 020	8.8	
Disability caused by a brain lesion	115 341	7.9	13 493	.9	128 834	4.4	
Hearing disability	45 492	3.1	34 312	2.4	79 804	2.7	
Visual disability	37 841	2.6	19 417	1.3	57 258	2.0	
Other	30 283	2.1	10 301	.7	40 584	1.4	

*Note*: Data are presented as *n* or %.

* *P*‐value for Chi‐square test.

Table 2 presents the prevalence of diagnosed comorbid diseases and survival between the groups. Hypertension was the most common condition diagnosed before inclusion in both the LTC and matched cohorts, followed by diabetes, dementia, osteoporosis and cerebral infarction. The matched cohort presented a similar pattern of disease distribution to that of the overall population, except for a significantly lower proportion of dementia. However, the LTC cohort had a different distribution of comorbidities; dementia and cerebral infarction were significantly more prevalent in the LTC cohort than in the matched cohort (43.8% vs 20.3% and 36.5% vs 19.9%, respectively, *P* < .0001). The proportion of all oral diseases diagnosed at the time of inclusion was higher in the matched cohort than in the LTC cohort. Periodontitis was the most commonly diagnosed in both the LTC and matched cohorts (54.2% and 63.8%, respectively).

**TABLE 2:** Prevalence of diagnosed comorbid diseases and survival between cohorts.

	Long‐term care cohort (*n* = 1 459 163)		Matched cohort (*n* = 1 459 544)		Total (*n* = 2 918 707)	
	*n*	*%*	*n*	*%*	*n*	*%*
*Medically diagnosed cases*						
Hypertension	959 891	65.8	939 534	64.4	1 899 425	65.1
Dementia	639 369	43.8	296 479	20.3	935 848	32.1
Cerebral infarction	532 755	36.5	290 442	19.9	823 197	28.2
Diabetes mellitus	503 936	34.5	456 196	31.3	960 132	32.9
Osteoporosis	415 766	28.5	455 090	31.2	870 856	29.8
Angina pectoris	216 355	14.8	232 955	16.0	449 310	15.4
Rheumatoid arthritis	106 992	7.3	108 717	7.4	215 709	7.4
Myocardial infarction	57 738	4.0	52 562	3.6	110 300	3.8
Erectile dysfunction	6914	.5	9322	.6	16 236	.6
*Dentally diagnosed cases*						
Impacted tooth	26 469	1.8	31 699	2.2	58 168	2.0
Dental caries	390 061	26.7	510 604	35.0	900 665	30.9
Other diseases of hard tissues of teeth	161 412	11.1	245.951	16.9	407 363	14.0
Pulpitis	533 787	36.6	689 271	47.2	1 223 058	41.9
Periodontitis	790 479	54.2	931 648	63.8	1 722 127	59.0
*Survival and death cases*						
Survival	410 556	28.1	865 574	59.3	1 276 130	43.7
Death	1 048 607	71.9	593 970	40.7	1 642 577	56.3

*Note*: Data are presented as *n* or %.

### 3.2 Population utilising dental services

**FIGURE 3:**
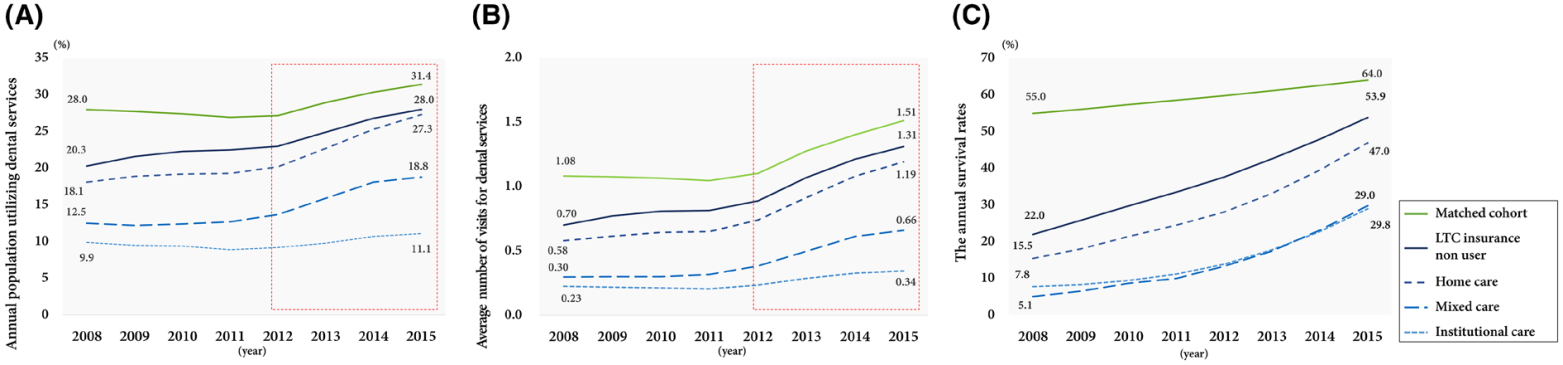
Dental service utilisation patterns and annual survival rates of each cohort from 2008 to 2015 reveal an upward trend in all (A) the annual population using dental services, (B) the average number of visits for dental treatment, and (C) the annual survival rates. With the exception of the institutional group, all cohorts exhibited a significant upward trend in dental service utilisation starting in 2012. This notable increase can be attributed to a change in national health insurance support. Given the similar increases in survival rates across all LTC groups, potentially attributable to concurrent social changes, including enhanced medical support, there arises a concern regarding the possibility of societal oversight regarding dental service utilisation among patients in institutional healthcare centers. [Colour figure can be viewed at wileyonlinelibrary.com]

The patterns of the population utilising dental services in all cohort groups are presented in Figure 3. The matched cohort had a higher proportion of recipients who received dental service utilisation in each year (around 30%), and the LTC cohort had less independence (LTC insurance non‐user > home care > mixed care > institutional care) and lower dental utilisation rates: 18.1‐27.3% for the LTC insurance non‐user and home‐care group, 12.5‐18.8% for the mixed‐care group and 10.7% for the institutional care group. The utilisation rate of dental services suddenly increased in 2012 for all groups except for the institutional care group. The patterns of average number of visits for dental services per person had similar patterns to the dental service utilisation rates.

### 3.3 Survival rate and dental service utilisation

Table 3 presents the survival and mortality during the observational period. The survival and mortality rates in the LTC cohort were almost twice those of the matched cohort (*P* < .0001): 28.1% survived and 71.9% died in the LTC cohort, and 59.3% survived and 40.7% died in the matched cohort. The survival rates of all LTC cohorts were significantly higher in dental service users than in the subgroups that received no dental services in the same cohort. The LTC insurance non‐user and home‐care groups had a survival rate three times higher than for those who received no dental services, and those for the institutional care and mixed‐care groups were twice as high.

**TABLE 3:** Survival rate in the long‐term care cohort according to dental service utilisation.

	Dental service utilisation						
	No			Yes			*P*‐value*
	Total	Death	Survival rate	Total	Death	Survival rate	
	*n*	*n*	%	*n*	*n*	%	
LTC insurance non‐user	126 550	114 951	9.2	279 260	184 669	33.9	<.0001
Home care	159 300	137 409	13.7	423 478	249 426	41.1	<.0001
Institutional care	87 671	79 541	9.3	116 708	88 787	23.9	<.0001
Mixed care	80 516	68 761	14.6	185 680	125 033	32.7	<.0001

*Note*: Data are presented as *n* or %.

* *P*‐value for Chi‐square test.

## 4 DISCUSSION

In this cohort study, we constructed a large‐scale LTC cohort of LTC insurance recipients and a matched cohort of South Korean patients during 2008‐2015 from the NHIS database, and investigated the patterns of dental service utilisation and survival rate in these cohorts. The main findings of this study were as follows: (1) the population of utilising dental services was significantly lower in the LTC cohort than in the matched cohort, which was observed to be more explicit in groups with more dependency, and (2) the survival rate for the LTC cohort was significantly higher among those who used dental services than in those who did not.

Patterns of the population utilising dental services reflect the need for oral care and the accessibility of dental clinics.^25^ The prevalence rates of periodontal destruction and periodontitis increase with age through a cumulative effect,^26^ and the risk of dental caries is affected by the extent of exposed tooth surfaces.^27^ Ageing processes influence salivation and hyposalivation can directly aggravate dental caries formation.^28^ Previous epidemiologic data have also indicated higher prevalence rates of dental caries and periodontal diseases in older patients.^29^ Moreover, various systemic diseases are either directly or indirectly correlated with oral diseases such as periodontitis.^30^, ^31^ Deterioration in oral health status was associated with a variety of health‐related adverse outcomes, including disability, neurodegenerative diseases such as dementia and Alzheimer's disease, and may result from a lack of resources (financial, human and social capital) needed in older age.^32^ While it is difficult to establish a clear causal relationship between accumulated oral diseases and chronic conditions over the lifespan, considering the life‐course journey in old age, the need for oral care and dental treatment among older people may increase as they gradually adapt and respond to worsening oral conditions. The need for oral care and dental treatment may therefore increase in older people.^33^ This is consistent with the present result of increased dental service utilisation in all groups except for the institutional care group.

However, most elderly recipients are vulnerable due to their physical frailty and receive financial support from family or society, and are therefore less likely to visit a dental office.^34^ Dental treatments are normally only performed in an outpatient clinic with specific equipment rather than at a normal visit to the doctor, and difficulties in accessibility to a dental office can restrict the availability of oral care to older patients. The present study also found significantly lower dental service utilisation in the LTC cohort, especially in the institutional care group. Considering that the grades of the LTC recipients are assigned according to care dependence, these results might indicate that higher dependency reduces the use of oral healthcare. This was consistent with previous findings that the oral health of care‐dependent older people was significantly worse than in older people who live independently.^35^, ^36^

The oral health of dependent people is generally poor, which negatively affects masticatory function and oral‐health‐related quality of life.^8^, ^37^ Numerous studies have found an association between systemic and oral diseases such as periodontitis.^38^ Cardiovascular diseases and cerebral infarction are the systemic diseases that are most significantly correlated with periodontal disease.^39^ Inadequate oral care can cause changes in the oral microflora, and may be a critical risk factor for life‐threatening conditions such as secondary cerebral attack or myocardial infarction in the LTC cohort, which had higher prevalence rates of various cardiovascular diseases. Especially for long‐term hospitalised patients, aspiration pneumonia is the disease most commonly related with mortality that can be affected by the oral condition or oral microflora.^40^ The systematic literature indicates that there is a significant association between oral hygiene and bacterial pneumonia, indicating the importance of understanding oral microbiome changes in older people in residential care in comparison with healthy individuals.^41^

Considering the risks for institutionalised patients associated with neglected oral hygiene, poor oral health is thought to be related to higher mortality indirectly by causing poor nutrition and eating behaviour.^42^ Since the risk of malnutrition in people who depend on care is as high as 90%, it is important to ensure adequate nutrition through oral healthcare and treatment.^43^ All of these factors can affect the survival rates of each cohort directly and indirectly, and all of the LTC cohorts in the present study had significantly higher survival rates in the subgroups who utilised dental service compared with those who did not in the same group. Regarding possible mechanisms underlying the relationship between oral health and mortality, many studies have found that maintaining or increasing oral function may be implicated in reducing the risk of mortality and of the development of frailty and other major adverse health‐related outcomes.^8^, ^44^ However, given that our study focused on older people and LTC insurance recipients, our findings should be interpreted with caution. The relationships observed may be influenced by a variety of factors, and the need for oral care and dental treatment should be understood within the broader context of each individual's overall health and life‐course journey.

In South Korea, dental treatment for oral diseases, excluding prosthodontic treatment, is covered by the public insurance system, allowing anyone to receive treatment based on their individual needs. This insurance coverage facilitates access to dental care regardless of geographical location. However, the system mainly requires patients to visit dental offices rather than offering home visits. This presents a significant barrier for LTC recipients, particularly those in institutional care, who may struggle to access dental treatment regularly and promptly. Our findings underscore the lower utilisation of dental services among LTC recipients, particularly evident in those with higher care dependency levels. This highlights the urgent need for policy considerations aimed at improving accessibility to dental care for vulnerable LTC populations.

Given that this study retrospectively establishes cohorts based on the national insurance database, it is imperative to recognise several limitations that affect the ability to draw definitive conclusions. First, it is essential to acknowledge that not all data pertaining to individuals within each cohort could be included, particularly detailed clinical information from individual medical records. Notably, 28% of the LTC cohort falls into the non‐user group, individuals who do not receive any public LTC support. While demographic differences were not observed between the user and non‐user groups, it is prudent to interpret all present data conservatively. Second, it's crucial to consider that each individual within the cohorts has a distinct follow‐up period, potentially influencing the survival rate. Lastly, while the extensive data from this cohort study demonstrates a notable correlation between dental service utilisation and the survival rate of LTC recipients, but there is a lack of information on survival by frequency and type of dental treatment. Additionally, our study includes individuals already have major disabilities and severe diseases, which adds complexity to understanding these relationships. Therefore, further studies involving fragmented data or the integration of real‐world clinical records are warranted to establish a more definitive causal relationship beyond the observed correlation.

## 5 CONCLUSIONS

Within the limitations of this study, the present large‐scale cohort data revealed that LTC recipients in South Korea experience social neglect for oral care as evidenced by lower utilisation of dental services; however, further study is needed to test the hypothesis that professional care in a dental office may significantly improve survival rates.

## AUTHOR CONTRIBUTIONS

K.A.K. analysed the data and drafted the manuscript; Y.T.K. and J.S.L. conceived ideas and critically reviewed the manuscript; B.A.L. collected and analysed the data.

## FUNDING INFORMATION

This work was supported by the National Health Insurance Service Ilsan Hospital grant (NHIMC2020‐20‐001), the National Research Foundation of Korea (NRF) funded by the Ministry of Science, ICT & Future Planning (NRF‐2022R1A2C2005537), and the Korea Health Technology R&D Project through the Patient‐Doctor Shared Decision Making Research center (PDSDM), funded by the Ministry of Health & Welfare, Republic of Korea (RS‐2023‐KH142251).

## CONFLICT OF INTEREST STATEMENT

The authors declare that they have no conflict of interest.

## Supporting information


Data S1:

Table S1:
