# Supplementary material for: Professional dental care and survival rates in long‐term care recipients: A cohort study of 1 459 163 individuals in South Korea
Source: Gerodontology. 2024 Jul 30;42(2):216–24. doi: 10.1111/ger.12781 (PMC12106944; doi:10.1111/ger.12781)
Supplement: Supplementary file 2 — Table S1: [file GER-42-216-s002.docx]

Supplementary Information

**Relationship between receiving professional dental care and the survival rate of long-term care patients: a cohort study of 1,459,163 long-term care beneficiaries in South Korea**

**Appendix Table 1. Distribution of long term care insurance participants**

|  | Level 1 | Level 2 | Level 3 | Level 4 | Level 5 | Level 6 | Out of grade A | Out of grade B | Out of grade C | Re-evaluation |  |
| --- | --- | --- | --- | --- | --- | --- | --- | --- | --- | --- | --- |
| Total (N.,%) | 244,271  (17) | 285,084  (20) | 458,151  (31) | 212,468  (15) | 19,715  (1) | 1,289  (0) | 61,405  (4) | 43,831  (3) | 16,259  (1) | 116,690  (8) |  |
| Gender | | | | | | | | | | | |
| Male | 79,888 | 92,468 | 154,647 | 57,768 | 4,718 | 287 | 20,400 | 14,460 | 6,279 | 42,365 |  |
| Female | 164,383 | 192,616 | 303,504 | 154,700 | 14,997 | 1,002 | 41,005 | 29,371 | 9,980 | 74,325 |  |
| Age | | | | | | | | | | | |
| ≤ 29 | 185 | 58 | 106 | 41 | 3 | - | 21 | 3 | - | 185 |  |
| 30-39 | 591 | 206 | 529 | 274 | 7 | - | 130 | 26 | 14 | 316 |  |
| 40-49 | 2,739 | 1,504 | 3,567 | 2,050 | 82 | 6 | 792 | 324 | 111 | 966 |  |
| 50-59 | 10,107 | 7,175 | 14,337 | 8,382 | 581 | 52 | 2,726 | 1,367 | 541 | 2,157 |  |
| 60-69 | 42,010 | 42,876 | 77,131 | 48,062 | 4,872 | 407 | 13,477 | 11,412 | 4,204 | 26,105 |  |
| 70-79 | 98,494 | 119,034 | 197,020 | 108,846 | 11,467 | 746 | 27,191 | 20,886 | 7,787 | 53,943 |  |
| 80 ≤ | 90,145 | 114,231 | 165,461 | 44,813 | 2,703 | 78 | 17,068 | 9,813 | 3,602 | 33,018 |  |
| Income | | | | | | | | | | | |
| First quintile | 67,654 | 80,594 | 139,228 | 70,036 | 7,098 | 596 | 23,372 | 20,168 | 9,153 | 44,069 |  |
| Second quintile | 22,264 | 26,502 | 42,853 | 19,408 | 1,710 | 97 | 5,647 | 3,596 | 1,188 | 10,822 |  |
| Third quintile | 30,017 | 34,895 | 55,853 | 24,723 | 2,175 | 129 | 7,234 | 4,376 | 1,326 | 13,573 |  |
| Fourth quintile | 43,928 | 51,384 | 80,012 | 35,489 | 3,217 | 177 | 9,670 | 5,948 | 1,800 | 18,453 |  |
| Fifth quintile | 80,408 | 91,709 | 140,205 | 62,812 | 5,515 | 290 | 15,482 | 9,743 | 2,792 | 29,773 |  |
| Presence of disability | | | | | | | | | | | |
| None | 168,093 | 205,281 | 331,998 | 156,556 | 15,855 | 1,014 | 44,465 | 33,486 | 12,241 | 91,567 |  |
| Present | 76,178 | 79,803 | 126,153 | 55,912 | 3,860 | 275 | 16,940 | 10,345 | 4,018 | 25,123 |  |
| Type of disability | | | | | | | | | | | |
| Non-disabled | 168,093 | 205,281 | 331,998 | 156,556 | 15,855 | 1,014 | 44,465 | 33,486 | 12,241 | 91,567 |  |
| Physical disability | 25,461 | 32,534 | 54,177 | 28,040 | 2,139 | 160 | 7,780 | 5,251 | 1,957 | 12,151 |  |
| Disability of Brain lesion | 33,932 | 27,107 | 34,452 | 11,252 | 406 | 22 | 3,114 | 1,318 | 406 | 3,332 |  |
| Hearing disability | 6,961 | 8,614 | 14,895 | 6,371 | 637 | 35 | 2,052 | 1,487 | 658 | 3,782 |  |
| Visual disability | 5,268 | 6,403 | 12,364 | 6,243 | 383 | 31 | 2,089 | 1,465 | 563 | 3,032 |  |
| Survival or Death cases | | | | | | | | | | | |
| Survival | 26,388 | 47,489 | 119,352 | 123,401 | 13,777 | 1,183 | 18,563 | 18,245 | 7,087 | 35,071 |  |
| Death | 217,883 | 237,595 | 338,799 | 89,067 | 5,938 | 106 | 42,842 | 25,586 | 9,172 | 81,619 |  |

**Appendix Table 2. Korea Informative Diseases Classification codes for Comorbid and dental disease included in the study**

| Disease | ICD-10 codes |
| --- | --- |
| Medical disease |  |
| Cerebral infarction | I63,I64,I65,I66 |
| Angina pectoris | I20 |
| Myocardial infarction | I21,22 |
| Hypertension | I10,I15 |
| Diabetes mellitus | E10,E11,E12,E13,E14,E15 |
| Rheumatoid arthritis | M05,M06 |
| Erectile dysfunction | N48,N49 |
| Osteoporosis | M80,M81,M82 |
| Dementia | F00 |
| Dental disease |  |
| Impacted teeth | K01 |
| Dental caries | K02 |
| Disease of hard tissue | K03 |
| Pulpitis | K04 |
| Periodontitis | K05 |

**Appendix Table 3. Korean Health Insurance Review and Assessment Service codes for dental treatment included in the study.**

| Dental treatment | Procedures | Code(s) |
| --- | --- | --- |
| Extraction | Extraction-Anterior Tooth | U4412 |
|  | Extraction-Posterior Tooth | U4413 |
|  | Extraction-Complicated Extraction | U4414 |
| Endodontic treatment | Access Cavity Preparation | U0050 |
|  | Pulp Extirpation | U0101 |
|  | Root Canal Enlargement | U0116, U0119 |
|  | Root Canal irrigation | U0111 |
|  | Root Canal Filling with Condensation Method | U0126 |
| Periodontal treatment | Scaling | U2232, U2233 |
|  | Root planning | U2240 |
|  | Subgingival curettage | U1010 |
| Implant surgery | Dental Implant-Diagnosis and Treatment Plan | UB111, UB112, UB113, UB114, UB115, UB116, UB117, UB118, UB119 |
|  | Dental Implant-Fixture Placement Operation | UB121, UB122, UB123, UB124, UB125, UB126, UB127, UB128, UB129 |
|  | Dental Implant-Prosthetic Restoration | UB131, UB132, UB133, UB134, UB135, UB136, UB137, UB138, UB139 |
| Denture | Resin Based Complete Denture | UA101,UA102,UA103,UA104,UA105,UA106  UA107,UA108,UA109,UA111,UA112,UA113,UA114,UA115,UA116,UA117,UA118,UA119,UA121,UA122,UA123,UA124,UA125,UA126,UA127,UA128,UA129,UA131,UA132,UA133,UA134,UA135,UA136,UA137,UA138,UA139,UA141,UA142,UA143,UA144,UA145,UA146,UA147,UA148,UA149 |
|  | Removable Partial Denture | UA301,UA302,UA303,UA304,UA305,UA306  UA307,UA308,UA309,UA311,UA312,UA313,UA314,UA315,UA316,UA317,UA318,UA319,UA321,UA322,UA323,UA324,UA325,UA326,UA327,UA328,UA329,UA331,UA332,UA333,UA334,UA335,UA336,UA337,UA338,UA339,UA341,UA342,UA343,UA344,UA345,UA346,UA347,UA348,UA349,UA351,UA352,UA353,UA354,UA355,UA356,UA357,UA358,UA359 |
|  | Metal Based Complete Denture | UA501,UA502,UA503,UA504,UA505,UA506  UA507,UA508,UA509,UA511,UA512,UA513,UA514,UA515,UA516,UA517,UA518,UA519,UA521,UA522,UA523,UA524,UA525,UA526,UA527,UA528,UA529,UA531,UA532,UA533,UA534,UA535,UA536,UA537,UA538,UA539, UA541,UA542,UA543,UA544,UA545,UA546  UA547,UA548,UA549 |

**Appendix Table 4. The number of people using dental service of each cohort from 2008 to 2015**

| Year | Matched cohort | | LTC insurance non user | | Home care | | Mixed care | | Institutional care | |
| --- | --- | --- | --- | --- | --- | --- | --- | --- | --- | --- |
|  | n | % | n | % | n | % | n | % | n | % |
| 2008 | 104707 | 28.0 | 42671 | 20.3 | 18187 | 18.1 | 652 | 12.5 | 5803 | 9.9 |
| 2009 | 157334 | 27.7 | 58220 | 21.6 | 39800 | 18.9 | 2313 | 12.2 | 6450 | 9.5 |
| 2010 | 180762 | 27.4 | 69885 | 22.3 | 44350 | 19.2 | 3937 | 12.4 | 7877 | 9.4 |
| 2011 | 188155 | 26.9 | 76838 | 22.5 | 43492 | 19.3 | 3072 | 12.7 | 9605 | 8.9 |
| 2012 | 198670 | 27.2 | 83619 | 23 | 44845 | 20.2 | 3425 | 13.7 | 11078 | 9.2 |
| 2013 | 224654 | 28.9 | 94446 | 24.9 | 55109 | 22.7 | 4425 | 15.9 | 12473 | 9.8 |
| 2014 | 251807 | 30.4 | 106922 | 26.8 | 67925 | 25.3 | 5003 | 18.1 | 14444 | 10.7 |
| 2015 | 276606 | 31.4 | 114187 | 28 | 81421 | 27.3 | 5621 | 18.8 | 16032 | 11.1 |

Data are presented as n or %

**Appendix Table 5. The number of visit for dental treatment of each cohort from 2008 to 2015**

| Year | Matched cohort | | LTC insurance non user | | Home care | | Mixed care | | Institutional care | |
| --- | --- | --- | --- | --- | --- | --- | --- | --- | --- | --- |
|  | Mean | SD | Mean | SD | Mean | SD | Mean | SD | Mean | SD |
| 2008 | 1.08 | 2.75 | 0.70 | 2.19 | 0.58 | 1.93 | 0.30 | 1.19 | 0.23 | 1.01 |
| 2009 | 1.08 | 2.74 | 0.77 | 2.33 | 0.61 | 1.99 | 0.30 | 1.21 | 0.22 | 1.02 |
| 2010 | 1.07 | 2.71 | 0.81 | 2.38 | 0.64 | 2.07 | 0.30 | 1.18 | 0.21 | 0.97 |
| 2011 | 1.05 | 2.70 | 0.82 | 2.42 | 0.65 | 2.09 | 0.32 | 1.25 | 0.21 | 0.98 |
| 2012 | 1.10 | 2.80 | 0.89 | 2.57 | 0.74 | 2.28 | 0.38 | 1.44 | 0.24 | 1.10 |
| 2013 | 1.28 | 3.12 | 1.07 | 2.92 | 0.92 | 2.65 | 0.50 | 1.71 | 0.29 | 1.32 |
| 2014 | 1.40 | 3.33 | 1.21 | 3.17 | 1.08 | 2.92 | 0.61 | 1.99 | 0.33 | 1.46 |
| 2015 | 1.51 | 3.50 | 1.31 | 3.33 | 1.19 | 3.09 | 0.66 | 2.13 | 0.34 | 1.49 |

Data are presented as mean or standard deviation
